# An argument for discovery-driven research: from physicist to cancer researcher

**DOI:** 10.3332/ecancer.2014.ed38

**Published:** 2014-07-03

**Authors:** Michael Retsky

**Affiliations:** Harvard School of Public Health, Boston, MA 02115, USA; Royal Free and UCL Medical School, Centre for Clinical Science and Technology, University College London, London, UK

My colleagues and I recently reported what may be an inexpensive and non-toxic method to prevent most if not all early relapses in breast and other cancers [1]. However, as a bizarre development, what could be a major medical advance turned into a researcher’s nightmare because with the current drug development business model there seems to be no way to conduct expensive confirmatory clinical trials of an inexpensive therapy.

This counterintuitive situation was brought to the attention of Pulitzer Prize winning reporter Jake Bernstein from ProPublica—a non-profit investigative journalistic organization. In the US and Europe, newspapers are being financially squeezed by news reporting on the internet. As a result, old fashioned investigative journalism is practiced much less frequently than in prior times. ProPublica was formed in an attempt to fill that gap. Bernstein investigated this situation and found that there are additional low cost treatment strategies that may also prevent relapse but are in the same financial predicament. Bernstein wrote a report on this issue that was published on their website [2]. My (only partially planned) personal role as a colon cancer patient and breast cancer researcher was used by Bernstein as an aid in discussing what have been named financial orphan cancer therapies. Since publication of the ProPublica report I have been asked on several occasions to explain in more detail how my roles as patient and researcher developed and how this relates to financial orphan therapies. I would like to take this opportunity to explain this improbable development.

I was trained as an experimental physicist at the University of Chicago. That fine school has a strong theoretical history and even experimentalists are expected to be able to use computations to augment their experiments. About eight years after getting my PhD, I was working at Hewlett-Packard in Colorado Springs, Colorado, when my good friend Robert Wardwell’s wife was diagnosed with gastric lymphoma. Wardwell knew medical oncologist Jack Speer for many years and wanted to do something to help his wife. He organized a small study group consisting of himself, me, Dr Speer and Victor Petrosky—another HP physicist.

We decided to make a computer simulation of breast cancer because there was a lot of data available for that cancer. Also I was using computer simulation at HP for my work and they graciously allowed access afterhours for community service applications. We naively thought this would be relatively straightforward. Speer told us that it was well known how tumors grow, how they are detected, and how they responded to chemotherapy. What we discovered however was quite different from our initial expectations. Our first paper in 1984 suggested breast cancer growth was intermittent with periods of dormancy rather than continuous or Gompertzian as previously thought [3]. This was quite important since the rationale for maximum tolerated chemotherapy for a period of months after surgery to prevent relapse was predicated on the idea that tumor growth was most rapid when the cancer was small and gradually growth slowed as the tumor became larger. Chemotherapy drugs are mostly effective when cancer cells are actively dividing.

This subject struck me as being very important and it also had a raw edge to it compared to the mature and staid physics research and engineering where I was quite skilled and could anticipate three or four ways to solve any new problem that arose.

I was a relative newcomer to HP and found that my work there was not particularly challenging. I was doing basic engineering work that was below my level of competency which gave me a lot of spare time in which to pursue my interest in cancer research. I liked living in Colorado Springs and especially liked the breast cancer research project, so as my job did not require my full attention to do it well, I was able to read the cancer literature and write cancer growth programs over a period of five years.

So, how can this research best be described? It would not be well described as hypothesis driven. I had no clear plan and did not know where it would lead. I had a vague notion that there was a problem with the conventional theory and was exploring the literature and writing programs. This would be best described as discovery based rather than hypothesis based. Research grants are only given for hypothesis driven research.

I had Speer to guide me and access to the well stocked medical library at Penrose Cancer Hospital. I was especially fascinated with the old literature that described how untreated cancers grew and how animals injected with rapidly growing multi-passaged cancer cells could be cured if and only if every cancer cell could be killed with toxic treatment.

Examining the reasoning behind maximum tolerated chemotherapy led me to Gompertzian kinetics and chasing down the evidence for Gompertzian kinetics led me to the Laird papers [4, 5]. Reading those papers in depth led me to discover that it was all based on one study that involved measuring tumor growth in only 18 rodents and one rabbit. Laird wrote “The pattern of growth defined by the Gompertz equation appears to be a general biological characteristic of tumor growth”. In addition there is a basic mathematical flaw repeated 19 times in the Laird papers. Remarkably, much of the experimental evidence for maximum tolerated chemotherapy that has been used with only partial success to prevent relapse on a very large number of patients is based on a single highly flawed study.

The Laird papers have been cited over 800 times by cancer researchers and physicians including key persons who formulated the basic concepts for maximum tolerated dosage adjuvant chemotherapy still often used today after surgery to remove primary tumors [6–11].

Speer later told me that I had a distinct advantage not being taught about cancer in a formal classroom setting. I had to read all the old papers myself rather than have someone in authority tell me how tumors grow.

This discovery never would have happened without the unusual situation with Wardwell and his wife, Speer, the unchallenging nature of my day job, and my solid background in science and fascination with cancer research. Turn a well-trained basic scientist loose with financial support, no prior knowledge of the subject and no particular hypothesis in mind. How is that for a research strategy? Try and get a grant to do something like that!

HP later wanted to downsize and offered voluntary severance with a generous package. I opted for that and got a position as Professor of Biology at University of Colorado and later moved to Connecticut with a company that marketed laboratory services in oncology. A few years later in a very melodramatic development, I was diagnosed with stage IIIc colon cancer. How strongly did I think I knew how tumors grow? Was I sure enough to actually use what I learned to design a therapy and if so what would that therapy look like? Would the quality of cancer research improve if the researcher had to live or die depending on the accuracy of his or her research?

I certainly did not plan it that way but knowing much about cancer before becoming a patient was a distinct advantage. I did not panic as I might have without this prior experience. I am sure I would not have taken the time to study old papers on tumor growth and make a logical decision to use low dose therapy rather than the usual high dose therapy. A cancer patient is inundated with information, much of which is unsolicited. Everyone has an opinion that he or she is more than willing to share. This information is offered with the best of intentions but provides much confusion at a time when clear thinking is essential. For example my nephew is a GI doctor and his advice was to take the conventional therapy first and then try something experimental. It was reasonable advice but I am sure that if I had taken six months of toxic treatment I would not want to do any more therapy. I had to ignore these suggestions that were offered with best of intentions.

As a general issue I would advise healthy persons to learn about lethal diseases before it happens that you get a sudden diagnosis. Familiarity at least to me was extremely helpful in making clear decisions.

I would like to conclude with a short discussion of what is now called metronomic chemotherapy [12–14]. After diagnosis in 1994, I started low dose 5-fluoruracil administered at 30% reduction from long term tolerable levels. This common colon cancer drug has been previously used this way to extend survival in late stage disease but never before to prevent relapse in early stage disease. This particular therapy was designed by Bill Hrushesky MD and I made some modifications. I have come to appreciate that it was an excellent design. Dosage used was 200 mg/m2 /day given over 5 hours usually from whenever I went to bed. The side effects were barely noticeable. My finger tips were somewhat numb and soft with cracks in my skin at finger joints and the nails grew in coarsely. I had a few blood blisters in my mouth but nothing else. My physician was monitoring white blood count but it never varied from normal. After a while he stopped that measurement. There was no physical discomfort whatsoever. I felt fine and was able to exercise daily. I considered the exercise as part of the protocol and continue to this day. Meanwhile the therapy involved a lot of fussing with flushing lines every morning and reprogramming the portable pump every evening. It was a general nuisance and I needed to decide when to quit. I went through some simple reasoning. First I assumed that the drug was perfect – i.e., any cancer cell that tried to divide while under the therapy would die. I also assumed that the cancer cells all divided once and only once in each volume doubling time – much like soldiers in a parade. Under those ideal conditions, how long should therapy continue? The answer was simple – one volume doubling time which for metastatic colon cancer is 2–3 months. In reality cancer cells are not so well behaved and the drug is less than 100% effective. Taking reasonable estimates, I calculated therapy needs to be longer than 2 years. That would be a long time to be on a toxic therapy. I doubt any patient could stand high toxicity for 2 years. So therefore the therapy must be non-toxic. Thus using simple reasoning, adjuvant chemotherapy to absolutely prevent relapse need be given over 2 years and must be non-toxic. That is approximately what I did. I stayed on therapy for 2.5 years.

## Conclusion

Needless to say, I did not relapse. I was on Judah Folkman’s staff at the time and discussed this therapy with him. Tim Browder was brought into the discussion. Browder later tested the therapy in mice and published the surprising result in 2000 that some common cancer drugs have antiangiogenic properties if used at low dose for extended times [12]. This is now called metronomic chemotherapy. Folkman and Browder are now deceased and I (the cancer patient) am the only one left alive to tell how metronomic chemotherapy began.

## Conflicts of Interest

M. Retsky has a patent pending for treatment of early stage cancer, is on the Board of Directors of Colon Cancer Alliance (www.ccalliance.org), and is Editor-in-Chief of the Journal of Bioequivalence and Bioavailability. No other conflicts of interest reported.
